# 

**Published:** 1863-06

**Authors:** 


### PHILADELPHIA DENTAL COLLEGE.

### FIRST ANNUAL SESSION, 1863-64.

### FACULTY.

C. A. KINGSBURY, D.D.S.,
Professor of Dental Physiology and Operative Dentistry.

THOS. WARDLE, D.D.S.,
Professor of Mechanical Dentistry and Metallurgy.

J. H. McQUILLEN, D.D.S.,
Professor of Anatomy, Physiology and Hygiene.

J. FOSTER FLAGG, D.D.S.,
Professor of Institutes of Dentistry.

HENRY MORTON, A.M.,
Professor of Chemistry.

GEO. W. ELLIS, D.D.S.,
Demonstrator of Operative Dentistry.

WM. GORGES, D.D.S.,
Demonstrator of Mechanical Dentistry.

The Laboratory of the College will be open, and preliminary lectures will be delivered by one of the Professors every day during the month of October; the lecture on Wednesday of each week, at 3 o'clock P.M., to be devoted to *Clinical* teaching. The regular Course of instruction will commence on the first Monday of November, and continue until the close of the ensuing February.

The lectures will be amply illustrated by the extensive and *valuable* collections of Anatomical, Pathological, and Mineralogical specimens, and the Philosophical and Chemical apparatus of the incumbents of the various Chairs, and every opportunity will be afforded in the Clinic and Laboratory for obtaining a *practical* knowledge of Operative and Mechanical Dentistry.

### FEES.

| | |
|---|---|
| Matriculation (paid but once) - - - - - | $5 00 |
| Tickets for the Course, including the Demonstrators' - | 100 00 |
| Diploma - - - - - - - - - - | 30 00 |

For further particulars, address J. H. McQUILLEN, DEAN,
July '63 1112 *Arch Street*, *Philadelphia.*

---

### Pennsylvania College of Dental Surgery.

### SESSION 1863-4.

### Faculty.

J. D. WHITE, D. D. S. Emeritus Professor.
T. L. BUCKINGHAM, D. D. S. Professor of Chemistry and Metallurgy.
C. N. PEIRCE, D. D. S. Prof. of Dental Physiology & Operative Dentistry.
E. WILDMAN, D. D. S. Professor of Mechanical Dentistry.
G. T. BARKER, D. D. S. Prof. of Principles of Dental Surgery and Therapeutics,
W. S. FORBES, D. D. S. Professor of Anatomy aud Physiology.
JAMES TRUMAN, D. D. S. Demonstrator of Operative Dentistry.
E. N. BAILEY, D. D. S. Demonstrator of Mechanical Dentistry.

The regular Course will commence on the first Monday of November and continue until the first of March ensuing.

During October the Laboratory will be open, and a Clinical Lecture delivered every Saturday, by one of the Professors, at 3 o'clock, P. M.

The most ample facilities are furnished for a thorough course of practical instruction.

Tickets for the Course, Demonstrators' Tickets included, $100. Matriculation Fee, $5. Diploma Fee, $30.

For further information, address, C. N. PEIRCE, *Dean.*

### Ohio College of Dental Surgery.

SESSION OF 1863–4.

Tho regular course of Lectures in this Institution, will commence on the first Monday of November, and close on the 20th of February.

### Faculty.

JAS. TAYLOR, D. D. S. 171 Race Street.
Institutes of Dental Science.

J. TAFT, D. D. S. 172 Race Street
Operative Dentistry.

GEO. EDWIN JONES, M. D. 481 West Seventh Street.
Anatomy and Physiology.

H. R. SMITH, D. D. S. 13 West Fourth Street.
Mechanical Dentistry.

H. A. SMITH, D. D. S. 118 West Sixth Street.
Chemistry and Metallurgy.

E. COLLINS, D. D. S.
Adjunct Prof. of Operative and Mehanical Dentistry.

**Text Books.**—Gray's and Wilson's Anatomy; Carpenter's, Todd and Bowman's Physiology; Williams' Pathology; Fown's, Turner's, Graham's, or Brand and Taylor's Chemistry; Taft's Operative Dentistry; Richardson's Mechanical Dentistry; Harris' Principles and Practice of Dental Science.

### Terms of Admission.

Tickets must be procured at the beginning of the session.

| | |
|---|---|
| Tickets for the course, - - | $100 00 |
| Matriculation Fee, - - | 5 00 |
| Diploma Fee, - - - - | 30 00 |

### Terms of Graduation.

Two courses, (including one of dissections,) with a dental pupilage.

Four years reputable practice will be received as equal to one course.

An acceptable thesis upon some subject on Dental science.

Must possess a good moral character, and be twenty-one years old.

For further information, address J. TAYLOR, *Dean.*
or J. TAFT, *Secretary.*

### CONTENTS.

### DENTAL REGISTER,

### OF THE WEST.

Issued Monthly, at $3 a year in advance.

DEVOTED TO THE INTERESTS OF THE DENTAL PROFESSION.

**Edited by J. TAFT and GEO. WATT.**

The Volume commences in January and closes in December.

The Register being issued monthly is always fresh, therefore indispensable to the practitioner who desires to keep posted up with the new inventions and improvements which are daily developed. Neither expense nor effort will be spared to give value and variety to its contents. Articles will be illustrated with well executed engravings, whenever they will add to the interest or assist in explanation.

Its extensive circulation and frequency of issue makes it the BEST DENTAL ADVERTISING MEDIUM in the United States.

☞ CONTRIBUTIONS SOLICITED.

Address Original Communications to **Geo. Watt, Xenia, O.**

Exchanges to **J. Taft**, or **Dental Register, Cincinnati, Ohio.**

Business Communications to

J. TAFT, Publisher,
172 Race Street, Cincinnati, O.

JNO. T. TOLAND, Proprietor,
38 West Fourth Street, Cincinnati, O.

Communications must reach the Editor as early as the 15th, to insure insertion in the next issue.

